# Differential Sources of Distress in Clinical and Research Trainees: A Focus on Work and Role Relationships

**DOI:** 10.1016/j.mayocpiqo.2025.100601

**Published:** 2025-03-19

**Authors:** Laurence M. Boitet, David A. Rogers, Katherine L. Sweeney, Mark C. Schall, C. Allen Gorman, Bobby Jones

**Affiliations:** a Department of Medical Education, University of Alabama at Birmingham, Birmingham, AL; b UAB Medicine Office of Wellness, University of Alabama at Birmingham, Birmingham, AL; c Department of Surgery, University of Alabama at Birmingham, Birmingham, AL; d Department of Sociology, University of Alabama at Birmingham, Birmingham, AL; e Department of Industrial and Systems Engineering, Auburn University, Auburn, AL; f Department of Management, Information Systems, and Quantitative Methods, University of Alabama at Birmingham, Birmingham, AL; g Department of Clinical and Diagnostic Sciences, University of Alabama at Birmingham, Birmingham, AL

## Abstract

**Objective:**

To examine the experience of clinical and research trainees within an academic medical center setting to identify and compare factors influencing their wellbeing.

**Participants and Methods:**

A cross-sectional, anonymous survey was conducted from June to July 2022 at a large academic medical center. Responses from clinical and research trainees were analyzed for this study. The survey assessed participant wellbeing using the Well-Being Index (WBI), in addition to perceptions of individual and organizational factors. Data were analyzed to identify correlates of elevated WBI scores and differences in clinical and research experience.

**Results:**

Ordinal logistic regression analysis identified low sense of recognition (odds ratio [OR], 0.59; 95% CI, 0.44-0.79), low role clarity (OR, 0.58; 95% CI, 0.42-0.80), moral distress (OR, 1.61; 95% CI, 1.24-2.10), and social isolation or loneliness (OR, 2.79; 95% CI 1.69-4.61) as significantly associated with high WBI scores, accounting for 48.8% of predicted variance. Additionally, distressed clinical trainees (WBI≥2) reported lower control over their work (*P*<.001) and significantly higher perceived stress from heavy workload (*P<*.05) and long hours (*P*<.01), compared with distressed research trainees. Distressed research trainees reported lower levels of trust in their supervisor (*P*<0.01), lower perceived organizational support (*P*<.05), and lower role clarity (*P*<.05), compared with distressed medical trainees. Low recognition, moral distress, and perceived stress from social isolation and loneliness were experienced similarly between groups.

**Conclusion:**

Our study indicates that trainees experience high levels of distress, although the sources differ. To effectively address these challenges, organizations should implement interventions targeted to address specific stressors of each group.

Two predominant trainee populations exist within academic medical centers: clinical and research trainees. Clinical trainees navigate educational programs that are designed to teach skills and knowledge necessary for patient care,^1^^,^^2^ whereas research trainees primarily study the mechanisms of human physiologic and behavioral processes that inform interventions applied to human health and disease.^3^ Although these workforces differ in training and focus, they are inextricably linked in their contribution to innovation in medicine.^3^ However, both fields face concerning trends, including declining applicant numbers^4^^,^^5^ and high turnover,^6^, ^7^, ^8^, ^9^ raising questions about the challenges trainees face in their training environments.

Clinical and research trainees operate within the same academic medical ecosystem, where common structural, cultural, and organizational factors shape their experiences. Distress among clinical trainees is well-documented and higher than that of most other student groups,^10^, ^11^, ^12^, ^13^ stemming from financial strain, intense study and practice requirements, high pressure, and lack of time for a quality social life.^12^ Although it is widely recognized that the experience of research trainees is considerably understudied,^14^, ^15^, ^16^ existing reports indicate that research trainees experience academic pressure and issues with supervisory relationships, in addition to high levels of burnout and mental health concerns.^17^^,^^18^ Although both groups are known to experience distress as a result of their training experience, their distinct career trajectories and training demands necessitate identifying common and unique correlates of distress.

A lens for understanding trainee distress is the Job Demands-Resources Model (JD-R), which posits that occupational wellbeing is determined by the balance between job demands and available resources.^19^ Experiencing job demands while provided appropriate resources is important for learning.^11^ When demands outweigh available resources, distress can occur, which is associated with burnout and low quality of life.^19^^,^^20^ Although clinical and research trainees navigate distinct roles, they share common organizational demands, including performance pressure, heavy workloads, and high expectations.^12^^,^^17^^,^^18^ Despite their training occurring in the same academic medical ecosystem, these trainees often have variable access to resources and minimal direct interaction, making it essential to examine both shared and distinct aspects of their experiences. Importantly, trainee wellbeing is protective and can prevent long-term burnout, improve academic productivity, prepare trainees for workplace stress, and improve patient care.^18^^,^^21^ Therefore, understanding the interplay between job demands and resources and their impact on the trainee experience is crucial for developing effective, evidence-based interventions tailored to specific trainee populations.

Despite increasing institutional efforts to implement wellbeing programs,^22^, ^23^, ^24^, ^25^ gaps remain in how to best support different trainee populations within the academic medical center. This study aimed to fill this gap by examining convergent and divergent factors that may contribute to trainee distress across clinical and research trainee groups. Using data from a large institutional employee engagement survey, this study seeks to lay the groundwork for the optimization of rigorous, targeted studies that inform the development of interventions that address upstream drivers of trainee distress. Although distress is common between trainee roles, we hypothesized that specific correlates of distress differ between groups owing to variations in job demands and available resources across programs. This is of importance at the organizational level because applying the same interventions across training pathways may be ineffective or even counterproductive.^26^, ^27^, ^28^ Identifying factors specific to individual trainee populations will allow the precise application of evidence-based interventions to mitigate adverse mental health outcomes and promote wellbeing.

## Participants and Methods

### Data Collection and Study Sample

An anonymous cross-sectional employee engagement survey was distributed to the employees of a large academic medical center in the southeastern United States from June 1-July 8, 2022. The survey aimed to capture the experience of the workforce working within a large, multisite medical enterprise, including a university hospital, large eye hospital, and children’s hospital, as previously described.^16^^,^^29^, ^30^, ^31^ Participants were required to provide consent to participate in the study. If consent was not obtained, participants were automatically exited from the survey. A total of 6466 participants completed the survey of 23,697 eligible employees. The data were retrospectively used for this study to broadly characterize differences in training experience between clinical and research trainees. As such, we narrowed the sample to only include those in trainee roles. Of an eligible 1620 trainees, 347 completed the survey (21.4%). Groups were combined into clinical (eg, medical student, resident, and fellow) and research (eg, graduate and postdoctoral) trainees. Of note, physician scientist (MD-PhD) students were also included in the sample, but due to low sample size, they were categorized as either clinical or research trainees, depending on their trainee role at the time of survey.

The engagement survey was developed as previously described to inform the institution with actionable data to effect change within the medical center setting, capturing employee experiences with their work.^30^ The Medallia Experience Management Software Platform was used to collect data. This study was reviewed and approved by the surveying institution’s institutional review board (IRB-300006629).

### Dependent Variable

We assessed trainee wellbeing using the expanded 9-item Well-Being Index (WBI), calculating composite scores as previously described, ranging from −2 (no/low distress) to +9 (high distress).^20^ For the purposes of further understanding differential correlates of distress between trainee groups, distress was defined as WBI composite scores ≥ 2, indicating high fatigue, burnout, low quality of life, and suicidal ideation.^16^^,^^20^

### Independent Variables

Independent variables chosen for this study were informed by extensive literature search, content expertise, and stepwise regression modeling. Items have been previously used to understand the experience of healthcare workers in this setting.^29^^,^^31^ Resilience was captured using the previously validated abbreviated 2-item Connor-Davidson Resilience Scale. Composite scores were generated and ranged from 0 (low resilience) to 8 (high resilience).^32^ Perceived organizational support was captured using the 3-item abbreviated scale, and composite scores were generated as previously described, ranging from 3 (low perceived support) to 15 (high perceived support).^33^^,^^34^

To minimize participant fatigue, we measured the following variables using single-item measures. We ensured that each question was asked in clear and unambiguous terms to capture the perception of each variable, appropriate for this exploratory study.^35^, ^36^, ^37^, ^38^ An item measuring perception of available resources was used from the National Institute for Occupational Safety and Health Worker Well-Being Questionnaire (WellBQ).^39^ Single item measures of perception of work control, sense of respect, work expectations, sense of belonging, sense of recognition, ability to bring up tough problems and issues, and trust and confidence in direct supervisor were collected and measured on a 5-point Likert scale (1=Strongly disagree, 5=Strongly agree). The frequency of moral distress was measured on a 6-point scale, in which 1=Never and 6=Almost every workday. These measures were previously validated in a healthcare setting using the Veteran’s Affairs All Employee Survey.^40^ Participants were also asked to identify their major work-related and nonwork-related stressors from a predetermined list generated with the collective input from over 25 clinical and nonclinical leaders.^29^ This comprehensive list included long hours, negative or toxic culture or work environment, increased responsibilities, job demands, or heavy workload, and social isolation or loneliness. Participants were asked to select the most concerning stressors during the past 3 months from each predetermined list. Participants who identified any of these variables as stressors were assigned a 1, whereas those not selected were assigned a 0, as previously described.^16^^,^^29^^,^^31^

### Data Analysis

Raw data were exported from the Medallia Survey Management platform and imported to Stata SE version 18 (StataCorp). Data were stratified by trainee role and ordinal variables were represented by mean and SD. Binary variables were expressed by frequency and percentage. To test the association between variables and trainee role, χ^2^, Fisher exact, and analysis of variance tests were used. To understand variables associated with elevated WBI scores in trainees, ordinal logistic regression modeling was performed. Odds ratio (OR), SD, and 95% CI were calculated. Variance inflation factor values were examined to ensure absence of multicollinearity in the model. Of note, the average variance inflation factor score was 1.73, indicating lack of collinearity. Dominance analysis was performed to understand the relative contribution of each variable to the overall variation in the model. To understand the differences in experience between clinical and research trainees with high distress (WBI≥2), WBI composite scores were recoded to a binary variable (0=No or low distress; scores between −2 and +1), 1=Distress, scores between +2 and +9).^16^^,^^20^ Data were expressed as described, and associations between variables and distress were tested.

## Results

Of the 1,620 eligible trainees, 347 completed the survey (21.4%). Cases with at least 1 missing variable were omitted, for a sample size of 250 (72%). A total of 115 participants were female (46%), with most participants aged between 25 and 34 years (68.8%) and White (52.8%). This representation is congruent with published characteristics of these trainee groups.^41^^,^^42^ Of the participants, 142 were clinical trainees (56.8%), and 108 (43.2%) were research trainees. A full demographic description of the sample can be found in Supplemental Table (available online at http://www.mcpiqojournal.org).

A descriptive summary of the trainee sample is represented in Table 1. We detected no significant differences between mean WBI composite scores between trainee groups. Significance was observed when testing the association between trainee role and individual resilience score (*P*<.05), control over work (*P*<.001), clarity of work expectations (*P*<.05), and trust and confidence in supervisor (*P*<.01). Further, stress derived from heavy workload (*P*<.001), negative or toxic culture or work environment (*P*<.001), and long hours (*P*<.001) were significantly associated with trainee role. All tested variables were significantly associated with WBI, except for individual resilience scores (*P*=.929).

**Table 1:** Sample Characteristics (n=250)

	Clinical trainees	Research trainees	Total sample	*P*^a^	*P*^b^
	n=142	n=108	n=250		
WBI Score (−2 to 9)	2.43 (2.8)	2.46 (2.9)	2.44 (2.9)	.54 (NS)	NA
Resilience Score (0-8)	6.37 (1.3)	6.08 (1.5)	6.25 (1.4)	.047∗	.929 (NS)
Perceived Organizational Support Score (3-15)	9.32 (2.6)	9.03 (2.96)	9.12 (2.8)	.20 (NS)	<.001∗∗∗
Moral distress	2.44 (1.3)	2.18 (1.3)	2.32 (1.3)	.23 (NS)	<.001∗∗∗
At my department or unit, I am treated with respect.	3.94 (0.88)	4.00 (0.93)	3.97 (0.90)	.88 (NS)	<.001∗∗∗
I have control over how my work is carried out.	3.01 (1.0)	3.83 (1.0)	3.37 (1.1)	<.001∗∗∗	<.001∗∗∗
I know exactly what is expected of me at work.	3.70 (0.83)	3.70 (1.1)	3.70 (0.96)	.014∗	<.001∗∗∗
I feel a sense of belonging at work.	3.77 (0.94)	3.72 (1.1)	3.75 (1.0)	.43 (NS)	<.001∗∗∗
I am satisfied with the recognition I receive for doing a good job.	3.30 (1.1)	3.28 (1.3)	3.29 (1.1)	.078ˆ	<.001∗∗∗
Members in my workgroup are able to bring up problems and tough issues.	3.63 (0.96)	3.50 (1.1)	3.58 (1.0)	.38 (NS)	<.001∗∗∗
I have trust and confidence in my direct supervisor.	4.05 (0.81)	4.01 (1.2)	4.03 (0.99)	.001∗∗	<.001∗∗∗
My organization is willing to extend resources in order to help me perform my job to my best ability (eg, training, supplies, equipment, funding).	3.52 (1.1)	3.29 (1.2)	3.42 (1.1)	.61 (NS)	<.001∗∗∗
Perceived stressors, frequency (%)					
Increased responsibilities, job demands, or heavy workload.	65 (45.8)	32 (29.6)	97 (38.8)	.009∗∗	<.001∗∗∗
Negative or toxic culture/work environment.	47 (33.1)	23 (21.3)	70 (28.0)	.040∗	<.001∗∗∗
Long hours	64 (45.1)	24 (22.2)	88 (35.2)	<.001∗∗∗	<.001∗∗∗
Social isolation or loneliness	55 (38.7)	46 (42.6)	101 (40.4)	.54 (NS)	<.001∗∗∗

Values are mean (SD) unless specified.

NA, not applicable; NS, not significant; WBI, Well-Being Index.

a ∗*P*<.05, ∗∗*P*<.01, ∗∗∗*P*<.001 vs trainee role.

b ∗*P*<.05, ∗∗*P*<.01, ∗∗∗*P*<.001 vs WBI composite score.

Table 2 represents the results of an ordinal logistic regression model wherein WBI composite score was used as the outcome variable. Research trainees reported higher WBI scores on average, compared with clinical trainees (OR, 1.78; *P*<.05). Factors associated with higher WBI scores on average included higher incidence of moral distress (OR, 1.61; *P*<.001), in addition to stress derived from heavy workload (OR, 1.70; *P*<.05), long hours (OR, 2.15; *P*<.01), and social isolation or loneliness (OR, 2.79; *P*<.001). On average, low sense of recognition (OR, 0.59; *P*<.001) and low role clarity (OR, 0.58; *P*<.001) were associated with higher WBI scores. The variables that contributed most highly to overall variance for WBI scores in trainees were sense of recognition (13.8%), role clarity (12.6%), moral distress (11.3%), and stress derived from social isolation or loneliness (11.1%).

**Table 2:** Ordinal Logistic Regression Predicting Well-Being Index Score in Trainees (n=250)

	Well-Being Index Composite Score (-2-9)					Rank	Standard dominance statistics
	OR	SE	95% CI		*p*		
Trainee role (Reference: Clinical Trainees)							
Research Trainees	1.78	0.49	1.041	3.058	.035∗	15	0.01
Resilience Score (0-8)	1.16	0.11	0.964	1.385	.12	16	0.004
Perceived Organizational Support Score (3-15)	0.96	0.06	0.848	1.078	.47	8	0.056
Moral distress	1.61	0.22	1.238	2.101	<.001∗∗∗	3	0.11
At my department or unit, I am treated with respect.	0.86	0.18	0.572	1.289	.46	9	0.056
I have control over how my work is carried out.	0.82	0.12	0.613	1.101	.19	7	0.057
I know exactly what is expected of me at work.	0.58	0.10	0.416	0.800	.001∗∗	2	0.13
I feel a sense of belonging at work.	0.89	0.14	0.651	1.210	.45	10	0.053
I am satisfied with the recognition I receive for doing a good job.	0.59	0.09	0.441	0.790	<.001∗∗∗	1	0.14
Members in my workgroup are able to bring up problems and tough issues.	1.24	0.18	0.938	1.649	.13	13	0.030
I have trust and confidence in my direct supervisor.	0.91	0.17	0.631	1.306	.60	6	0.063
My organization is willing to extend resources in order to help me perform my job to my best ability (eg, training, supplies, equipment, and funding).	0.95	0.13	0.730	1.238	.71	12	0.042
Perceived stressors							
Increased responsibilities, job demands, or heavy workload	1.70	0.43	1.042	2.782	.034∗	11	0.050
Negative or toxic culture/work environment	0.87	0.24	0.500	1.501	.61	14	0.017
Long hours	2.15	0.56	1.286	3.599	.004∗∗	5	0.074
Social isolation or loneliness	2.79	0.71	1.694	4.607	<.001∗∗∗	4	0.11
Model fit							
Pseudo-*R*^*2*^	0.175						
AIC	1020.10						
BIC	1115.19						

AIC, Akaike Information Criteria; BIC, Bayesian Information Criteria; OR, odds ratio.

ˆ ∗*P*<.05, ∗∗*P*<.01, ∗∗∗*P*<.001.

Table 3 illustrates the relationship between distress and the measured variables, stratified by trainee role. Of note, distress is defined as WBI≥2, associated with risk for high fatigue, burnout, low quality of life, and suicidal ideation.^16^^,^^20^ Distressed clinical trainees reported a lower sense of control over their work (*P*<.001), and significantly higher frequency of stress derived from a heavy workload (*P*<.05) and long working hours (*P*<.01), compared with research trainees. Distressed research trainees reported significantly lower perceived organizational support scores on average compared with clinical trainees (*P*<.05). Further, these trainees reported lower clarity around work expectations (*P*<.05) and lower trust and confidence in their supervisor (*P*<.01) compared with clinical trainees. Notably, no significant differences between distressed trainee role categories for sense of recognition, moral distress, or stress derived from social isolation or loneliness were observed, indicating similar experiences of these variables across role categories.

**Table 3:** Description of Variables by Incidence of High Distress (WBI≥2) (n=150)

	Distress by trainee role		*P*
	Clinical (n=88)	Research (n=62)	
Resilience Score (0-8)	6.22 (1.2)	6.21 (1.3)	.93 (NS)
I have trust and confidence in my direct supervisor.	3.83 (0.83)	3.56 (1.3)	.002∗∗
My organization is willing to extend resources in order to help me perform my job to my best ability (eg, training, supplies, equipment, funding).	3.28 (1.0)	2.92 (1.1)	.21 (NS)
Moral distress	2.75 (1.3)	2.60 (1.5)	.34 (NS)
At my department or unit, I am treated with respect.	3.68 (0.92)	3.65 (0.99)	.97 (NS)
Perceived Organizational Support Score (3-15)	8.85 (2.5)	7.84 (2.7)	.021∗
I have control over how my work is carried out.	2.74 (0.98)	3.52 (1.1)	<.001∗∗∗
I know exactly what is expected of me at work.	3.43 (0.81)	3.21 (1.2)	.017∗
I feel a sense of belonging at work.	3.50 (0.98)	3.35 (1.1)	.42 (NS)
I am satisfied with the recognition I receive for doing a good job.	2.89 (1.0)	2.63 (1.2)	.14 (NS)
Members in my workgroup are able to bring up problems and tough issues.	3.40 (1.0)	3.19 (1.1)	.63 (NS)
Perceived stressors, frequency (%)			
Increased responsibilities, job demands, or heavy workload	50 (56.8)	25 (40.3)	.047∗
Negative or toxic culture/work environment	38 (43.2)	20 (32.3)	.18 (NS)
Long hours	51 (58.0)	21 (33.9)	.004∗∗
Social isolation or loneliness	45 (51.1)	33 (53.2)	.80 (NS)

Values are mean (SD) unless specified.

NS, not significant.

ˆ ∗*P*<.05, ∗∗*P*<.01, ∗∗∗*P*<.001 vs trainee role.

## Discussion

Our findings reinforce previous research indicating that clinical and research trainees face significant distress, relative to the general population.^43^, ^44^, ^45^, ^46^ This study builds on this in that it identifies key differences in potential sources of distress between these groups of trainees, underscoring the need for targeted interventions that address their unique challenges. By closely examining the nuances of trainee experiences, institutions can better implement proactive strategies that mitigate distress and foster supportive environments conducive to both professional development and institutional success.

In this study, we described trainee wellbeing within the same academic medical center. This investigation was initiated within the same setting, wherein the ecologic contribution to distress (eg, general institutional environment) were static, to isolate specific factors contributing to distress between trainee groups. Both clinical and research trainees are engaged in rigorous training programs, but their structures and demands differ considerably. Clinical trainees follow a standardized, patient-care focused curriculum with predictable progression, whereas research trainee coursework varies widely depending on concentration. Similarly, clinical trainee assessment is highly standardized and related to curriculum content, whereas research trainee assessment is based on progress in research projects and publications. In this study, in the total sample of trainees, we detected no significant difference in mean WBI composite scores between groups, suggesting potential commonalities in their experience of distress. Overall, elevated WBI composite scores were significantly associated with a low sense of recognition, low role clarity, and increased frequency of moral distress, in addition to perceived stress derived from social isolation or loneliness. Importantly, we also observed that research trainees had higher odds of elevated WBI composite scores on average, compared with clinical trainees.

Our study further identified several key factors associated with distress among trainees. JD-R posits that job demands contribute to distress, whereas resources buffer against it.^47^ Our results align with this model, demonstrating that the perception of a heavy workload and long hours were significantly associated with elevated WBI scores. Moral distress was also significantly associated with elevated WBI scores, which we suggest acts as an emotional demand that creates cognitive burden. Our study also identified that a deficit in resources, specifically a low sense of recognition and perception of unclear work expectations, were among the strongest factors associated with distress. Notably, stress related to social isolation or loneliness was significantly associated with higher WBI scores, indicating a deficit in social resources fostering connection among trainees. These findings suggest that institutional policies should prioritize balancing available resources to meet job demands, especially given their impact on trainee wellbeing.

A low sense of recognition was significantly associated with higher WBI scores on average and contributed most to the variance in elevated WBI scores across the total trainee sample. Although there is scant literature on recognition practices in these trainee populations, research in other fields suggests that recognition acts as a resource, enhancing motivation, job satisfaction, and performance.^48^ We observed no significant difference between perception of recognition between trainee groups, suggesting that a low sense of recognition is a shared challenge. Mentors and supervisors should explore the application of recognition practices and alternative methods of accomplishing this, such as practices informed by individual preference.^49^ This may include the idea of existential recognition, including regular communication of goals and strategies, decisional involvement, involvement in the design and direction of projects, and available access to training and advancement.^50^

Role clarity was another notable correlate of distress, particularly among research trainees. Our findings reveal that although research trainees reported greater control over their work compared with clinical trainees, those experiencing high distress were markedly more likely to report unclear expectations. This ambiguity may stem from the unstructured nature of research training, where progress is often self-directed and assessment criteria are less standardized compared with those in clinical training.^51^ Given that role clarity is linked to lower incidence of burnout^52^ and improved innovation,^53^ institutions should enhance mentorship structures and provide clarity around expectations for research trainees. Mentors and supervisors should engage in ongoing, structured collaboration with trainees to identify work modifications that align with trainee interests and demands that might hinder progress. Our research team has previously found that this proactive approach to work modification leads to a decrease in overall distress among healthcare leaders.^54^

Moral distress correlated markedly with distress across both trainee groups. Although moral distress has been well-documented in clinical trainees,^55^, ^56^, ^57^ our findings suggest that it is also prevalent among research trainees. However, its specific manifestations remain underexplored, although moral distress within the academic setting has been described.^58^, ^59^, ^60^ We suggest that moral distress creates a cognitive and emotional demand, which has a negative consequence on trainee wellbeing. For clinical trainees, moral dilemmas stem from sources such as ethical disagreements with attendings, discriminatory patient care, and resource limitations.^55^^,^^57^ In contrast, the research trainee experience with moral distress is likely different and may include pressure to publish, authorship disputes, and supervisory misconduct. Moral dilemmas are commonly experienced in both clinical and research settings.^61^^,^^62^ It is therefore important for organizations to promote curriculum that enhances moral development and mature decision-making in trainees. Moral development teaches the ability to manage conflict, uncertainty, and value different perspectives,^63^ which are skills required for science and medicine.^62^^,^^64^, ^65^, ^66^, ^67^ Institutions should consider providing educational resources on ethical decision-making, conflict resolution, and resilience-building strategies to better equip trainees in navigating morally challenging situations. In addition, fostering open dialog and support through mentorship and peer networks may help mitigate the negative effects of moral distress.

Although clinical and research trainee mean WBI composite scores were similar, the differences in potential sources of distress between trainee groups suggest that interventions must be tailored according to trainee role. Clinical trainees reported a higher frequency of stress derived from increased responsibilities, job demands, or heavy workloads, and long hours, in addition to considerably less control over their work, compared with research trainees. Distressed research trainees reported significantly less trust and confidence in their supervisor, lower perceived organizational support scores on average, and lower clarity around work expectations, compared with clinical trainees. These data suggest that clinical trainees derive distress from their work demands, whereas research trainees derive distress from their relationship with their work and lack of tailored resources and support. As such, strategies to mitigate these sources of distress must strategically differ to meet the needs of specific trainee populations. For clinical trainees, addressing workload perceptions and control may be best achieved using transparent conversations and incorporating trainee input on curriculum structure and function. The experience of research trainees differs significantly, particularly in their interactions with those who influence their work. Mentoring relationships are known to be among the most important influences on doctoral trainee experience,^68^^,^^69^ partly because of the role of the mentor in trainee psychosocial and career support.^70^ Strengthening resources for developing robust mentoring relationships may aid in augmenting supervisor trust and perception of organizational support. Moreover, strong mentoring relationships could help reduce role ambiguity by prioritizing clear expectations and reciprocal, structured feedback.

The implications of this study must be considered within its limitations. Owing to its nature as a cross-sectional survey, causality cannot be determined, and generalizability is limited. Although the correlates of higher WBI distress scores can be interpreted within the context of trainee roles, the data were captured at a single institution, and participation was voluntary, introducing the possibility of nonresponse bias. Most of the sample was White and female, limiting our ability to assess factors contributing to distress that are related to identity. This study aimed to capture broad differences in trainee experience within the academic medical center; therefore, trainee roles spanning the entire training arc were combined into 2 broad categories: clinical and research. We acknowledge that organizational experiences likely vary across training stages within each designation, with distinctions between junior and senior trainees, as well as among master’s students, precandidacy and postcandidacy doctoral students, and postdoctoral fellows. Exploring these nuanced, role-specific differences will be a key focus of future research. In addition, although dual degree-seeking (ie, MD-PhD) trainees were included in the sample, they were categorized on the basis of their trainee role at the time of survey. Therefore, the MD-PhD trainee experience cannot be interpreted by this study, although we hypothesize it to differ from the other trainee populations. The threshold for determining distress used in this study is indicative of that of the general US working population.^20^ Future studies will determine the specificity for WBI to detect low quality of life, high fatigue, and suicidal ideation specific to different categories of trainees within the academic medical center. Data were collected as a part of an employee engagement survey, limiting investigator ability to use more expansive measures, and instead, single-item measures were used to maximize participant response and retention. Although interpretability of the responses may be limited, they do provide tangible insights into trainee experience in the applied setting. Further, these items were chosen from the National Institute for Occupational Safety and Health Worker Wellbeing Questionnaire and the Veteran’s Affairs All Employee Survey, with practical application in healthcare settings.^71^^,^^72^ Although there is evidence that single-item measures offer comparable predictive power with multiitem scales,^73^ we acknowledge that single items limit the validity and robustness of data interpretation; however, the data presented in this study are meant to draw attention to the differences in trainee perception of experiences that may contribute to distress. Although trainee role designations were included in the survey, we hypothesized that the 21.4% response rate was at least partly due to trainees not feeling recognized for their role within the organization. Despite these limitations, this study aimed to demonstrate that factors that may influence elevated WBI scores may be specific to individual institutions and therefore warrant careful attention across organizations.

## Conclusion

In conclusion, our study underscores the importance of recognizing the distinct challenges faced by clinical and research trainees within academic medical centers. By leveraging the JD-R model as a guiding framework, we suggest that approaches to mitigating distress in trainees require exploration into sources of distress. Leaders of training programs should consider the balance between job demands and available resources in training environments and programs. Future research should rigorously explore the experiences of trainees at different points in clinical and research training programs to better understand the unique demands associated with each stage. Addressing these issues proactively will not only improve trainee wellbeing but also contribute to a healthier, more resilient future workforce in medicine and research.

## Potential Competing Interests

The authors report no competing interests.

## Ethics Statement

This study was reviewed and approved by the surveying institution’s institutional review board (IRB-300006629). Participants were required to provide consent to participate in the study. If consent was not obtained, participants were automatically exited from the survey.
