# Supplementary material for: Differential Sources of Distress in Clinical and Research Trainees: A Focus on Work and Role Relationships
Source: Mayo Clin Proc Innov Qual Outcomes. 2025 Mar 19;9(2):100601. doi: 10.1016/j.mayocpiqo.2025.100601 (PMC11979368; doi:10.1016/j.mayocpiqo.2025.100601)
Supplement: Supplemental Table [file mmc1.pdf]

Supplemental Table. Participant Demographics

	Frequency(%)
Total Respondents	250(100)
Trainee Role	
Medical	142(56.8)
Biomedical	108(43.2)
Gender	
Male	98(39.2)
Female	115(46.0)
Non-binary or Self-describe	1(0.40)
Prefer not to answer	21(8.40)
Missing	15(6.0)
Race	
White	132(52.8)
Black or African American	12(4.8)
Hispanic or LatinX	9(3.6)
Asian or Pacific Islander	31(12.4)
Middle Eastern	7(2.8)
Multiracial	5(2.0)
Prefer not to answer	37(14.8)
Missing	17(6.8)
Age (category)	
18-24	20(8.0)
25-34	172(68.8)
35-44	23(9.2)
45-54	0(0.0)
55-65	2(0.8)
Prefer not to answer	17(6.8)
Missing	16(6.4)
